# Functional Redundancy Enables Stable Treatment Performance Across Taxonomically Diverse Dairy Wastewater Microbiomes

**DOI:** 10.1111/1758-2229.70448

**Published:** 2026-10-20

**Authors:** Alex Batista Trentin, Sara Letícia Stolfo dos Santos, André Felipe Bendix, Laila Cristina Lopes, Thaís Fernandes Mendonça Mota, Samara Silva de Souza, Nédia de Castilhos Ghisi, Deborah Catharine De Assis Leite

**Affiliations:** ^1^ Graduate Program in Agronomy Purdue University West Lafayette Indiana USA; ^2^ Graduate Program in Biotechnology (PPGBIOTEC) Universidade Tecnológica Federal Do Paraná (UTFPR) Dois Vizinhos Brazil; ^3^ Graduate Program in Computer Technologies for Agribusiness (PPGTCA) Universidade Tecnológica Federal Do Paraná (UTFPR) Medianeira Brazil

**Keywords:** dairy wastewater microbiome, functional redundancy, microbial community structure, predicted metabolic pathways, wastewater treatment ecology

## Abstract

Biological treatment of dairy wastewater depends on complex microbial communities that vary widely across reactors, influent sources and operating conditions. Whether stable treatment performance arises from conserved taxa or from redundancy in functional potential remains unclear. In this study, we conducted a meta‐analysis and reprocessing of 239 samples from nine independent studies spanning six countries and multiple reactor configurations. Taxonomic profiles showed extreme heterogeneity, with no ASV detected in at least 50% of samples and most genera exhibiting strong study‐specific patterns. In contrast, the predicted functional repertoire was highly conserved. PICRUSt2 analysis identified 196 MetaCyc pathways present in at least 90% of samples, and these pathways accounted for approximately 90% of total predicted functional abundance. Generalised linear latent variable models showed that temperature and pH created consistent but modest ecological gradients, while study‐ and reactor‐level factors dominated community structure. Despite substantial taxonomic turnover, functional signatures associated with fermentation, biosynthesis, nitrogen cycling, and degradation of complex substrates remained stable across systems. Treatment‐stage transitions also followed predictable metabolic trajectories. These results show that dairy wastewater treatment systems are taxonomically flexible but functionally robust, and that extensive redundancy supports consistent metabolic capacity across diverse operational and environmental contexts.

## Graphical abstract

Across diverse dairy wastewater systems, microbial communities exhibit strong taxonomic variability yet converge on a conserved set of metabolic pathways. This functional redundancy underpins process stability and consistent treatment performance despite differences in reactor design, influent composition and microbial community structure.

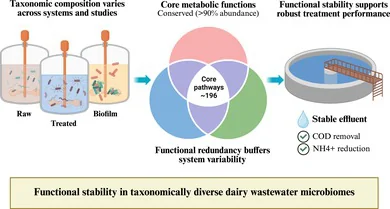

## 1 Introduction

The dairy industry is ubiquitous worldwide, with an average annual production of 900 million tonnes of milk. This activity can generate wastewater volumes approximately 2.5 times greater than the volume of processed milk, amounting to 2.25 billion tonnes annually (Ramsuroop et al. 2024). Effluents from this industry originate from various manufacturing stages and are characterised by a high organic load due to residues of milk, fats, proteins and lactose, as well as a complex combination of nutrients such as nitrogen and phosphorus. Furthermore, they may contain a range of chemicals used in equipment cleaning, including detergents and disinfectants (Alalam et al. 2021; Ankoliya et al. 2023). This compositional complexity results in a significant environmental impact, driving extensive research into the treatment and management of dairy wastewater (Bella and Rao 2023; Kaur 2021).

The high nutrient and organic content of dairy wastewater support a rich and diverse microbial community capable of degrading lactose, proteins and lipids through distinct metabolic pathways (Joshiba et al. 2019; Kolev Slavov 2017). Identifying the bacterial community present in the effluent allows for the elucidation of mechanisms involved in the degradation of its compounds (Irazoqui et al. 2022). Previous studies have examined the microbiome of dairy wastewater at the scale of individual treatment systems, revealing diverse microbial taxa associated with fermentation, organic matter cycling, and the degradation of recalcitrant compounds (Callejas et al. 2019; Giordani et al. 2021; Paulo et al. 2020). However, these studies were limited to specific operational conditions, and none systematically reprocessed raw sequencing data across multiple independent datasets under a unified bioinformatic pipeline. As a result, cross‐study comparisons remain restricted by methodological heterogeneity, and the conservation of microbial community structure and function across geographically and operationally diverse dairy wastewater systems remains not fully characterised.

Although microbial community diversity can be influenced by specific treatment methods and wastewater composition (Callejas et al. 2019; Palanisamy et al. 2022), certain microbial taxa frequently remain consistent across different treatment conditions, forming a core community with minimal variation (Gil‐Pulido et al. 2018; McAteer et al. 2020; Tsitouras et al. 2023; Zieliński et al. 2021). The core microbiome is commonly defined in taxonomic terms, emphasising the consistent detection of taxa across systems. Yet taxonomic consistency alone may not be sufficient to explain why treatment performance remains relatively stable under fluctuating operational conditions and highly variable influent compositions.

Evidence from other microbial ecosystems suggests that functional redundancy, whereby phylogenetically distinct taxa perform similar metabolic functions, may offer an additional perspective on the mechanisms that support system resilience beyond taxonomic composition alone (Martínez‐Álvaro et al. 2021; Toju et al. 2020). If functional redundancy operates in dairy wastewater systems, it would explain how treatment stability persists despite taxonomic variability: different bacterial assemblages could maintain equivalent metabolic capacity for lactose fermentation, protein hydrolysis and nutrient cycling. However, whether this concept extends to dairy wastewater treatment systems and how functional potential may be maintained despite taxonomic variability has not been systematically assessed across diverse studies.

The present study addresses these gaps through a meta‐analysis and comprehensive reanalysis of publicly available 16S rRNA amplicon datasets from dairy wastewater systems spanning multiple countries, reactor configurations and treatment strategies. Unlike previous single‐system investigations, this study integrates data from independent studies that sequenced different hypervariable regions of the 16S rRNA gene (including V1–V2, V3–V4 and V4–V5) and processed them under a unified bioinformatic pipeline to ensure cross‐study comparability. Beyond taxonomic characterisation, functional potential is explicitly evaluated to test whether core metabolic pathways are conserved despite taxonomic variability across systems. The objectives of this study are therefore to determine the taxonomic and functional core of the dairy wastewater microbiome, to identify the environmental and operational drivers of community composition, and to evaluate whether functional redundancy, rather than taxonomic stability, underpins the resilience and consistent performance of biological dairy wastewater treatment.

## 2 Methods

### 2.1 Study Selection and Data Acquisition

A systematic meta‐analysis of dairy wastewater microbiome studies was conducted by querying the Web of Science, Scopus and NCBI Sequence Read Archive (SRA) databases with the search terms: (‘dairy’ OR ‘milk’ OR ‘cheese’) AND (‘wastewater’ OR ‘effluent’ OR ‘anaerobic digestion’) AND (‘16S’ OR ‘microbiome’). The studies were selected based on the following inclusion criteria: (1) bacterial communities analysed via 16S rRNA gene amplicon sequencing; (2) samples from dairy wastewater (raw or from treatment plants); (3) publicly available raw sequence data; and (4) basic operational metadata. The studies screening information, and analysis workflow, are described in Figures S1 and S2, respectively. After selecting studies with relevant information, the raw FASTQ files were downloaded using the SRA Toolkit v3.0.0 (prefetch and fastq‐dump), and metadata were extracted from bioprojects and published articles.

Metadata were extracted from original publications and NCBI SRA BioSample records and standardised across studies. Variables included: Bioproject, conditions of the treatment, identification of DNA or cDNA sequencing, country where the samples were collected, type of treatment, origin of the inoculum, origin of the samples, type of wastewater, temperature, Chemical oxygen demand (COD), pH, different treatments used (biological, chemical or physical), type of reactor when present, agitation and digestion conditions.

Temperature was categorised as psychrophilic (< 20°C), mesophilic (20°C–45°C), or thermophilic (> 45°C), with continuous values retained. Treatment type was classified as Biological, Physical, Physical + Biological, Physical + Chemical + Biological, or None (untreated), with individual treatment components recorded separately. COD (mg L^−1^) and pH were standardised, with outliers (> 3 SD) flagged for verification. pH was additionally classified as acidic, basic, or neutral. Operational parameters (reactor type, agitation, batch/cycle designation, digestion type), temporal data (days since reactor start), and sample characteristics (origin, effluent type, country) were recorded when available. Missing values were coded as NA and not imputed. The full generated metadata sheet is available as (Supporting Information S1).

### 2.2 Sequence Processing and Quality Control

Raw sequences were processed independently for each study in QIIME2 (v. 2024.10) (Bolyen et al. 2019) using the q2‐dada2 plugin. Study‐specific processing was used to account for differences among datasets in sequencing quality, primer choice, and targeted 16S rRNA regions, thereby avoiding the introduction of artefacts associated with joint denoising across heterogeneous studies. Quality profiles were visualised using qiime demux summarise to guide parameter selection. Sequences were truncated based on the quality of each dataset sequencing; all the dada2 parameters used are available in Table S4. Chimeric sequences were removed using the consensus method, and amplicon sequence variants (ASVs) were inferred using the DADA2 algorithm (Callahan et al. 2016). Taxonomy was assigned using a naïve Bayes classifier trained on SILVA 138.2 (Chuvochina et al. 2026) (99% full‐length sequences) via the q2‐feature‐classifier plugin with a confidence threshold of 0.7, consistent with the default setting in QIIME 2 and with commonly adopted practice in previous microbiome studies using this classifier (Bokulich et al. 2018; Kaehler et al. 2019). ASVs that were unclassified at the Domain level, or assigned to Archaea, Eukaryota, chloroplasts, or mitochondria were removed from downstream analyses. Genus‐level unclassified ASVs were retained with the designation ‘Unclassified [Family]’.

Due to the variation in 16S rRNA regions across the studies (spanning V1–V2, V3–V4 and V4–V5 regions), ASVs generated from different studies cannot be directly compared through sequence alignment or clustering, since sequences covering distinct genomic intervals are not equivalent units of diversity. To resolve this and enable cross‐study comparability a reference‐based phylogenetic tree was constructed using SEPP (SATé‐enabled phylogenetic placement) (Mirarab et al. 2011) implemented through the q2‐fragment‐insertion plugin in QIIME2 (Bolyen et al. 2019). SEPP inserts short query sequences (ASVs) into a pre‐computed backbone phylogeny (SILVA 138.1), providing computational efficiency for large datasets while maintaining phylogenetic accuracy. The SEPP algorithm proceeded as follows: (1) decomposition of the backbone tree into overlapping subsets of approximately 50–100 sequences; (2) alignment of each query sequence to its most likely subset using HMMER; (3) phylogenetic placement calculation using pplacer to determine likelihoods across branches; (4) insertion at the maximum‐likelihood branch; and (5) ensemble combination of placements across all subsets. After the raw sequencing analysis, the feature table, representative sequences, taxonomy, and rooted tree were extracted for downstream analysis in R.

### 2.3 Data Filtering and Statistical Analysis

Post‐processing was conducted in R (version 4.3.0) using the phyloseq package (version 1.44.0). Quality filters were applied to remove ASVs with fewer than 10 total reads across all samples, ASVs present in fewer than two samples, and samples with fewer than 1000 reads. The initial dataset contained 19,186 ASVs across 239 samples. After filtering, the final dataset comprised 7512 ASVs across the same 239 samples, with a median sequencing depth of 45,832 reads per sample (interquartile range: 28,441–67,293). Data were not rarefied for community‐composition, ordination, and functional‐inference analyses because rarefaction discards valid reads and reduces statistical power. Instead, compositionally aware methods and relative‐abundance transformations were used as appropriate. Because richness‐based estimators are more sensitive to sequencing depth, observed richness, Chao1, and Faith's phylogenetic diversity were additionally evaluated in a rarefied sensitivity analysis.

Alpha‐diversity metrics were calculated in R using the phyloseq, vegan and picante packages. On the non‐rarefied count table, observed richness, Chao1 richness, Shannon diversity, and Simpson diversity were estimated for each sample. Because richness‐based metrics are more sensitive to sequencing depth, a rarefied sensitivity analysis was additionally performed, in which observed richness, Chao1 richness, Shannon diversity, Simpson diversity, and Faith's phylogenetic diversity were recalculated. Differences across studies were assessed using the Kruskal–Wallis test, with significance set at *p* < 0.05. To assess effect consistency and control for batch effects, within‐study Kruskal–Wallis tests were performed for studies with multiple temperature levels (*n* = 3) or treatment types (*n* = 7). Effects were considered robust if they were globally significant and either consistent across two or more studies (within‐study *p* < 0.10) or significant in the largest study (*p* < 0.05). ASV prevalence was calculated as the percentage of samples in which each ASV was detected. ASVs were classified as rare (prevalence < 10%) or core (prevalence ≥ 50%). The number of taxa meeting core criteria was determined at each taxonomic rank. The prevalence of selected taxa across studies was visualised as heatmaps, showing the percentage of samples containing each taxon within each study.

For multivariate community analysis, ASVs with valid genus‐level assignments, defined as non‐missing and non‐empty genus labels after taxonomy curation, were aggregated to the genus level. Although the complete dataset comprised 239 samples, only 160 were retained for modelling, due to filtering methods: (i) samples with complete metadata for both pH and temperature, which were the environmental covariates used in the constrained models, and (ii) genera that passed the prevalence filter (≥ 5% of samples). Samples lacking pH or temperature metadata, or containing zero genera after prevalence filtering, were excluded prior to model fitting to ensure a consistent dataset across all ordinations and to reduce instability associated with sparse or incomplete observations. Community structure was then analysed using generalised linear latent variable models (GLLVMs), which are model‐based ordination methods that jointly analyse counts across multiple taxa while simultaneously estimating the effects of measured environmental variables and the residual latent structure (Korhonen et al. 2025). A negative binomial distribution was specified because microbiome count data are discrete and typically overdispersed, with variance exceeding the mean (Zhang et al. 2017). This framework was selected instead of traditional distance‐based ordination methods because it directly models taxon counts, accommodates the mean–variance structure characteristic of amplicon sequencing data, and allows environmental effects to be estimated while accounting for unobserved sources of variation and study‐level batch effects. Three GLLVMs with two latent variables were fitted using the gllvm package (v1.4.3): (i) an unconstrained model, (ii) an environmental model including temperature and pH as predictors and (iii) a study‐corrected model including Study as a fixed effect to account for batch variation.

Model parameters were estimated using the optim (The R Core Team 2025) function with the BFGS algorithm, a quasi‐Newton optimisation method that iteratively updates parameter estimates to maximise the model likelihood (Andrei 2018). By using gradient information to approximate the curvature of the likelihood surface, BFGS can improve the efficiency and stability of parameter estimation in complex models (Dai 2013). This optimizer was selected because it provided stable convergence for the present dataset, avoiding the convergence problems observed with alternative settings. Candidate models were compared using the Akaike Information Criterion (AIC), and the model with the lowest AIC was considered the best‐supported model. Environmental effects were represented by arrows in ordination plots, indicating their direction and strength.

Marginal effects of predictor variables were assessed using permutational multivariate analysis of variance (PERMANOVA) with 999 permutations to estimate the variance associated with Study, Reactor type, Effluent type, Treatment type, Temperature, and Sample origin using the vegan package. To distinguish unique from shared sources of variation, variation partitioning was additionally performed on the genus‐level community matrix using the same complete‐case subset retained for multivariate modelling. Genera passing the prevalence filter were Hellinger‐transformed prior to analysis. Explanatory variables were grouped into three predictor blocks: study identity (Study), operational variables (Reactor type, Treatment type, Digestion, Effluent type and Sample origin), and environmental variables (temperature and pH). Adjusted *R* ^2^ values were used to estimate the variance uniquely attributable to each block and the variance shared among blocks, and the significance of unique fractions was evaluated using partial redundancy analysis with 999 permutations. All statistical tests were two‐tailed with significance at *p* < 0.05.

### 2.4 Functional Potential Prediction

Metabolic pathway prediction was performed using PICRUSt2 (v2.5.2) (Douglas et al. 2020), with default parameters configuration, to infer functional gene content from 16S rRNA amplicon data. Gene family abundances were predicted for each ASV using expanded ancestral‐state reconstruction, an evolutionary approach inferring the likely gene‐family content of taxa by using the gene content of phylogenetically related reference genomes across the placement tree (Langille et al. 2013). Prediction quality was assessed using the nearest‐sequenced taxon index (NSTI), which measures the phylogenetic distance between each ASV and its closest sequenced reference genome (Louca, Doebeli, and Parfrey 2018). ASVs with NSTI > 2.0 (PICRUSt2 default) were excluded because such distant placements have limited reference‐genome support and are therefore expected to yield less reliable functional predictions. This filtering step reduced the influence of poorly represented taxa and improved the interpretability of downstream pathway inference. Predicted gene families were mapped to MetaCyc (Caspi et al. 2018) metabolic pathways (v27.0) using MinPath (Ye and Doak 2009), and pathway abundance tables were generated using the pathway_pipeline.py script with default parameters. Pathway abundances were normalised to relative abundances to account for differences in sequencing depth.

Pathway richness was defined as the number of pathways per sample with relative abundance greater than 0.0001%. This low‐abundance threshold was used to distinguish detected pathways from near‐zero background values after relative‐abundance normalisation. For differential abundance testing, only pathways present in more than 10% of samples were analysed, to reduce the influence of highly sparse features and improving the robustness of group comparisons. Shannon diversity was computed for each sample using the vegan package (v2.6–4) (Oksanen et al. 2001). The core functional repertoire was defined as pathways present in ≥ 90% of samples with mean relative abundance > 0.001%. Pathway variability across samples was assessed using the coefficient of variation (CV = *σ*/*μ*), and pathways were classified as stable (CV < 0.5), moderately variable (0.5 ≤ CV ≤ 1.0), or highly variable (CV > 1.0). These categories were used as an operational summary of relative pathway stability rather than as formal inferential thresholds, based on different microbiome studies (Flores et al. 2014; Galloway‐Peña et al. 2017; Vandeputte et al. 2021).

Differences in functional diversity among sample types (biofilm, raw wastewater and treated wastewater) were assessed using Kruskal‐Wallis tests, followed by pairwise Dunn's post hoc tests with the Benjamini‐Hochberg correction when significant differences were detected (*α* = 0.05). Correlations between pathway richness and Shannon diversity were evaluated using Spearman's rank correlation (*ρ*). For differential abundance analysis, pathways present in > 10% of samples were tested using Kruskal‐Wallis tests. This approach was chosen over model‐based tools such as DESeq2 or ALDEx2 because PICRUSt2 pathway outputs are already relative‐abundance normalised continuous values and do not carry the integer count properties those models assume (Hawinkel et al. 2020). Effect sizes were calculated as eta‐squared ($\eta 2=\left(H-k+1\right)/\left(n-k\right)$) and interpreted as small (*η* ^2^ < 0.06), medium (0.06 ≤ *η* ^2^ < 0.14), or large (*η* ^2^ ≥ 0.14) (Gustafson et al. 2024). *p* values were adjusted using the Benjamini‐Hochberg false discovery rate (FDR) method, and pathways with FDR‐adjusted *p* < 0.05 were considered significantly differentially abundant. Pairwise comparisons between sample types were conducted using Wilcoxon rank‐sum tests with FDR correction. Log_2_ fold changes were calculated as log_2_ [(mean abundance in group A + 1 × 10^−6^)/(mean abundance in group B + 1 × 10^−6^)]. To visualise differentially abundant pathways, relative abundances were *z*‐score normalised and hierarchical clustering was applied to pathways. Alternative distance‐linkage combinations were evaluated using cophenetic correlation, and Euclidean distance with complete linkage was retained because it yielded the highest cophenetic correlation (*r* = 0.859) and therefore best preserved the original pairwise dissimilarity structure. All analyses were performed in R (v4.3.1) using base statistical functions and ggplot2 (v3.4.3) for visualisation.

## 3 Results

### 3.1 Dataset Overview and Sample Characteristics

The integrated dataset comprised 239 samples from 9 independent studies spanning 6 countries (Uruguay, Luxembourg, USA, Ireland, Canada, Italy and South Korea), representing diverse dairy wastewater treatment systems (Table 1). Ireland contributed the most samples (48.3%), followed by Canada (18.3%), Luxembourg (11.6%), Uruguay (10%), South Korea (4.7%), the USA (3.8%) and Italy (3.3%). Samples originated from treated wastewater (*n* = 121, 51%), raw wastewater (*n* = 117, 49%) and biofilms (*n* = 4, < 2%).

**TABLE 1:** Summarisation of the projects used in the analysis, with sample number and main information of wastewater type and treatment (when present).

Project	Country	Samples	Main wastewater type	Main treatment type	References
PRJNA1068640	Uruguay	24	Cleaning dairy wastewater	Biological	(Illarze et al. 2024)
PRJNA356222	Luxembourg	28	Dairy wastewater	Physical + Biological	(Henriet et al. 2017)
PRJNA385730	USA	9	Dairy wastewater	Biological	(Lai et al. 2018)
PRJNA601960	Ireland	48	Synthetic wastewater	Physical + Biological	(McAteer et al. 2020)
PRJNA616223	Ireland	24	Synthetic wastewater	Physical + Biological	(Trego et al. 2021)
PRJNA667972	Canada	44	Dairy wastewater	None reported	(Alalam et al. 2021)
PRJNA681387	Italy	8	Dairy wastewater	Physical + Biological	(Lembo et al. 2020)
PRJNA762671	South Korea	11	Dairy wastewater	Physical + Biological	(Kim et al. 2022)
PRJNA826074	Ireland	44	Dairy wastewater	Physical + Chemical + Biological	(Logan et al. 2022)

Effluent composition varied across studies, with 146 samples (61%) from dairy wastewater processing facilities, 72 samples (30%) from synthetic wastewater mimicking dairy effluent composition, and 24 samples (10%) from cleaning operations in dairy facilities. Treatment systems included biological reactors (*n* = 156, 65%), untreated raw wastewater (*n* = 68, 28%) and stabilisation lagoons (*n* = 18, 7%). Treatment complexity ranged from no treatment (PRJNA667972, *n* = 44) to combined physical, chemical and biological processes (PRJNA826074, *n* = 44). Reactor types included up‐flow anaerobic sludge blanket reactors (UASB, *n* = 60), expanded granular sludge bed reactors (EGSB, *n* = 24), sequencing batch reactors (SBR, *n* = 64) and granular sludge tank reactors (GSTR, *n* = 8).

Temperature regimes varied widely across systems, with mesophilic conditions (20°C–45°C) being the most common (*n* = 137, 57%), followed by psychrophilic conditions (< 20°C, *n* = 52, 22%) and thermophilic conditions (> 45°C, *n* = 9, 4%). Most samples were analysed using DNA‐based sequencing (*n* = 184, 76%), with cDNA (active community profiling) comprising 24% (*n* = 58), as shown in Table S1.

### 3.2 Alpha Diversity Patterns Across Studies

Shannon diversity varied significantly across studies (Kruskal–Wallis *χ* ^2^ = 153.9, df = 8, *p* < 0.001), with values ranging from 0.64 to 5.38 and a global median of 3.54 (CV: 35%) (Figure 1A). Simpson diversity showed a similar pattern (*χ* ^2^ = 135.2, df = 8, *p* < 0.001), indicating that differences in alpha diversity were consistent across non‐rarefied diversity metrics. In the rarefied sensitivity analysis, observed richness, Chao1 richness, and Faith's phylogenetic diversity also differed significantly across studies (Observed: *χ* ^2^ = 167.7; Chao1: *χ* ^2^ = 162.0; Faith's PD: *χ* ^2^ = 146.5; all FDR < 0.001), supporting the same overall biological interpretation. Rarefaction did not alter the main study‐level patterns, as sample‐level correlations between non‐rarefied and rarefied metrics were extremely high (*ρ* = 0.991–1.000), and study‐level rank concordance was perfect across all shared metrics (*ρ* = 1.000). PRJNA681387 (Italy, thermophilic GSTR reactor treating dairy wastewater, *n* = 8) exhibited the lowest diversity (median: 2.43). Studies with low‐to‐intermediate diversity included PRJNA385730 (USA, mesophilic lagoons treating dairy wastewater, *n* = 9, median~2.5), PRJNA667972 (Canada, untreated dairy wastewater, *n* = 44, median 1.41), and PRJNA616223 (Ireland, psychrophilic/mesophilic reactors treating synthetic wastewater, *n* = 24, median 3.82).

**FIGURE 1:**
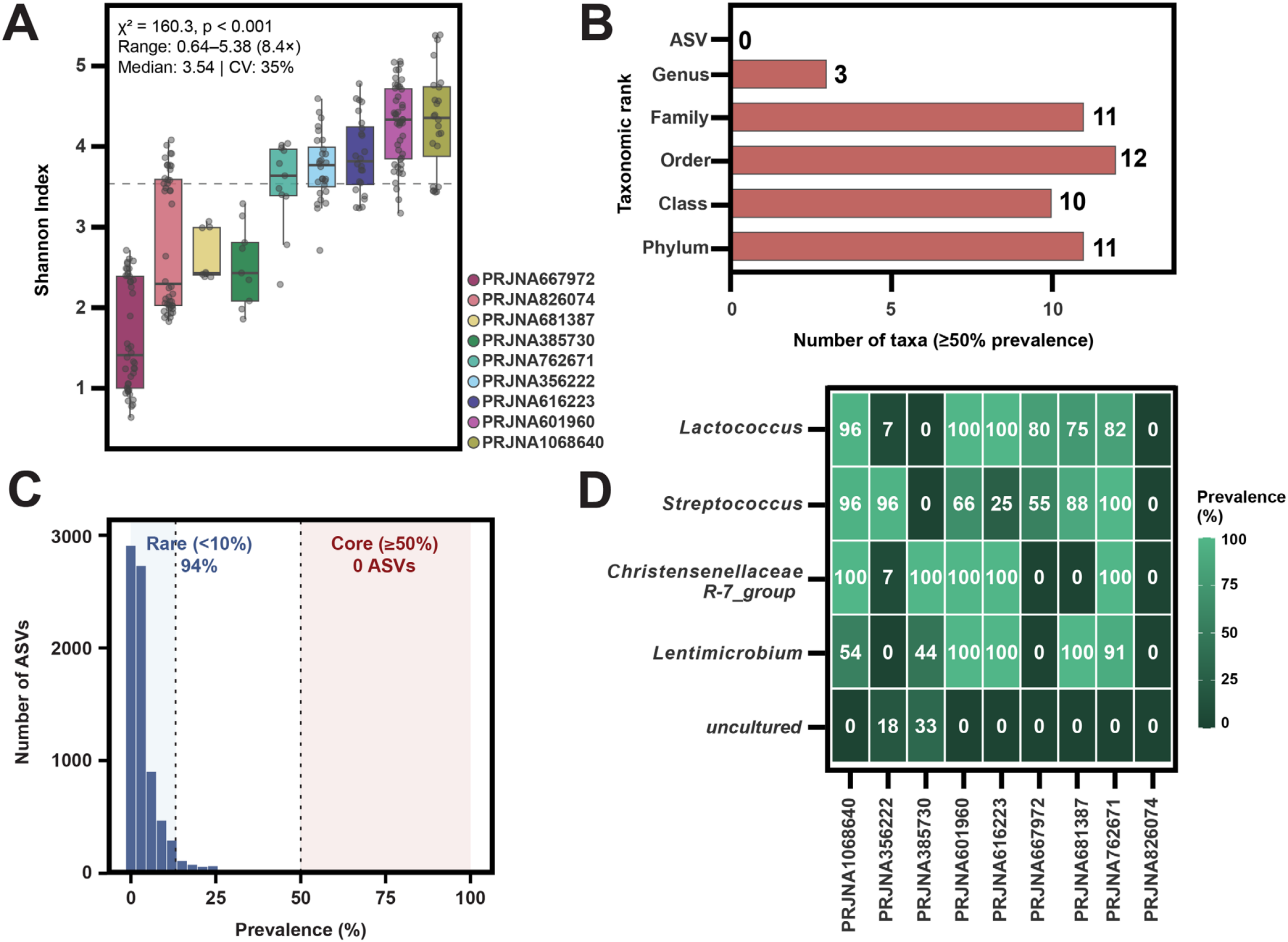
Taxonomic diversity and core microbiome across dairy wastewater systems. (A) Shannon diversity across nine studies showing significant inter‐study variation (Kruskal‐Wallis, *p* < 0.001). (B) Number of taxa at ≥ 50% prevalence by taxonomic rank. (C) ASV prevalence distribution; 94% of ASVs were rare (< 10% prevalence). (D) Prevalence of top five genera across studies (%).

Moderate‐to‐high diversity was observed in PRJNA356222 (Luxembourg, mesophilic SBR reactors treating dairy wastewater with varying treatments, *n* = 28, median *H*′≈3.8), PRJNA1068640 (Uruguay, psychrophilic and mesophilic lagoon systems treating cleaning wastewater, *n* = 24, median≈4.3), and PRJNA762671 (South Korea, mesophilic reactors treating dairy wastewater, *n* = 11, median≈3.6). The highest diversity occurred in PRJNA601960 (Ireland, psychrophilic and mesophilic UASB and EGSB reactors treating synthetic wastewater, *n* = 48, median≈4.3) and PRJNA826074 (Ireland, multi‐stage treatment system including physical, chemical and biological steps, *n* = 44, median≈2.29, SD≈0.83). The substantial inter‐study variation in diversity, spanning an 8.6‐fold range in median values, exceeded within‐study variability.

No ASVs were present in at least 50% of all 239 samples (Figure 1D), and 94% showed less than 10% prevalence across the dataset. Taxonomic aggregation to higher ranks increased core representation, yielding 3 core genera, 11 core families, 12 core orders, 10 core classes and 11 core phyla (Table S1), corresponding to approximately 0%–11% of taxa per rank (Figure 1B). Prevalence patterns varied widely among studies (Figure 1C). *Lactococcus* and *Streptococcus* were highly prevalent in most datasets but absent from PRJNA826074, whereas the Christensenellaceae R‐7 group reached 100% prevalence in several datasets but was absent in others. *Lentimicrobium* was strongly represented in PRJNA601960, PRJNA616223, PRJNA681387 and PRJNA762671 but nearly absent in the remaining studies.

### 3.3 Environmental Drivers of Community Composition

Generalised linear latent variable models (GLLVMs) were fitted to 703 genera across 160 samples from six studies (PRJNA1068640, PRJNA356222, PRJNA385730, PRJNA601960, PRJNA681387, and PRJNA826074) to disentangle environmental effects from study‐level batch structure. The remaining 79 samples lacked complete temperature and pH information and were therefore excluded from the GLLVM analysis. The unconstrained model (AIC = 232,717.5) revealed strong study‐driven structure in latent ordination space (Figure 2A). PRJNA826074 (Ireland, multi‐stage physical, chemical, and biological treatment) formed a distinct cluster separated primarily along latent variable 1, while PRJNA601960 (Ireland, synthetic wastewater, UASB/EGSB) occupied an intermediate region of ordination space. Outside the clearly separated PRJNA826074 cluster and the intermediate placement of PRJNA601960, only PRJNA385730 and PRJNA1068640 showed noticeable partial overlap in latent ordination space, while PRJNA681387 and PRJNA356222 remained more distinct, suggesting some shared compositional signatures between PRJNA385730 and PRJNA1068640 despite their geographic separation.

**FIGURE 2:**
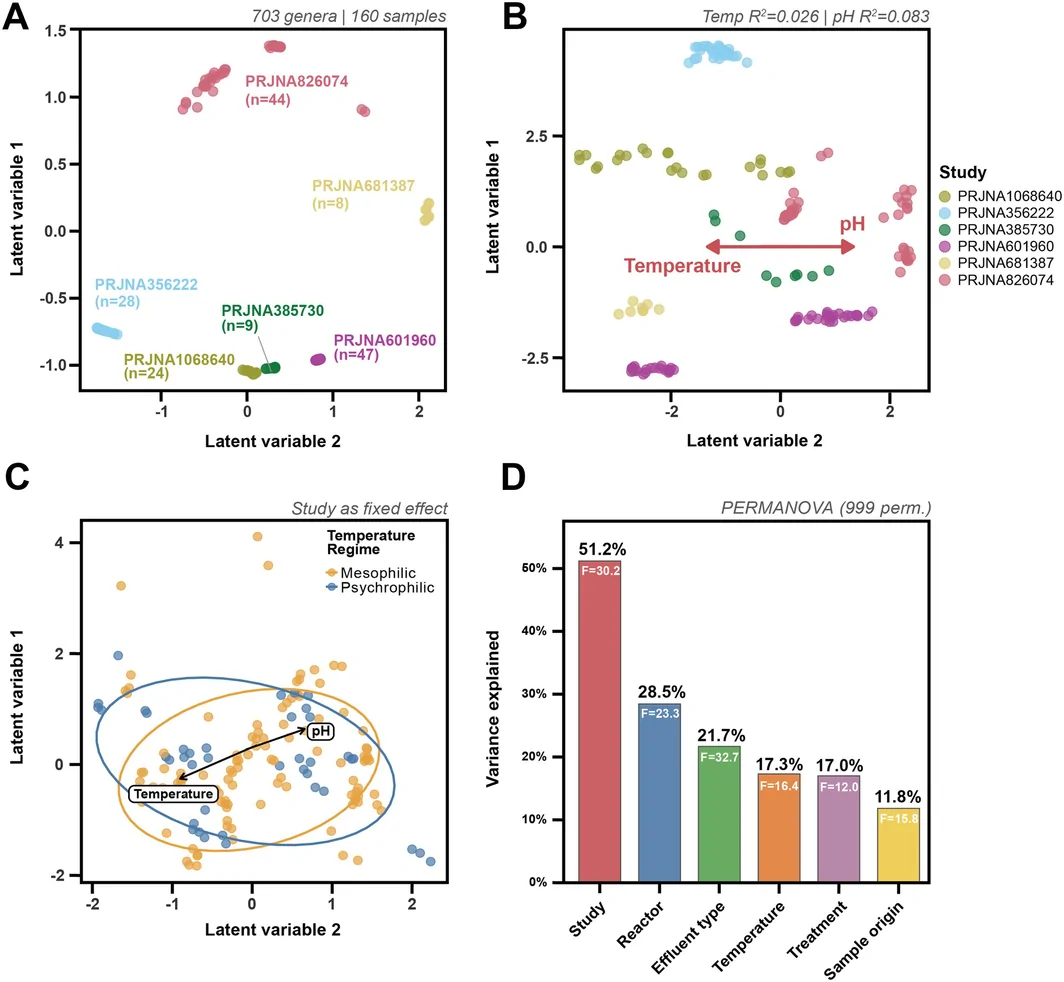
Drivers of microbiome community structure. (A) Unconstrained GLLVM ordination showing study‐specific clustering. (B) Environmental GLLVM with temperature and pH gradients. (C) Study‐corrected ordination coloured by temperature regime. (D) PERMANOVA variance partitioning; study explained 51.2% of variation.

The environmental model, which incorporated standardised temperature and pH, had substantially higher AIC (AIC = 244,716.1) compared with the unconstrained model (ΔAIC = +11,998.6), indicating that environmental predictors alone cannot account for the dominant sources of variation. Nonetheless, temperature and pH exerted measurable but modest effects on community structure, explaining 2.6% (*R* ^2^ = 0.026) and 8.3% (*R* ^2^ = 0.083) of compositional variation, respectively (Figure 2B). The approximate orthogonality of temperature and pH vectors indicates that these variables impose independent ecological constraints. The study‐corrected model, which included Study as a fixed effect together with temperature and pH, provided the best overall fit (AIC = 226,688.3), improving substantially over both the unconstrained and environmental models (Figure 2C). After accounting for study‐level effects, temperature‐related separation remained visually apparent along latent variable 1 in the study‐corrected GLLVM ordination. Quantitatively, temperature regime explained 17.3% of the compositional variation in the complementary PERMANOVA analysis (*R* ^2^ = 0.173, *p* = 0.001), supporting temperature as an important, though secondary, driver of community structure relative to study identity.

Permutational multivariate analysis of variance (PERMANOVA) corroborated the GLLVM results (Figure 2D, Table S2). Study identity was the strongest marginal predictor of community composition (*R* ^2^ = 51.2%, *F* = 30.17, *p* = 0.001), consistent with the pronounced study‐level separation observed in the ordinations. Among the remaining predictors, reactor configuration explained 28.5% of the variance (*F* = 23.27, *p* = 0.001), followed by effluent type (21.7%), temperature regime (17.3%), treatment type (17.0%), and sample origin (11.8%), indicating that both operational and environmental factors significantly structure dairy wastewater microbiomes. Because these predictors are not independent, we further applied variation partitioning on the 160‐sample complete‐case subset used for multivariate modelling. This analysis showed that the full model explained 64.4% of the genus‐level variation, of which 5.28% was uniquely attributable to study identity, 5.36% to operational variables, and 1.75% to environmental variables; all three unique fractions were significant (Study: *F* = 11.82, *p* = 0.001; Operational: *F* = 3.86, *p* = 0.001; Environmental: *F* = 4.59, *p* = 0.001). The remaining explained variation was largely shared among predictor blocks, indicating substantial overlap between batch, process, and environmental structure in the dataset.

### 3.4 Functional Potential and Core Pathways

PICRUSt2 analysis predicted 425 MetaCyc pathways across the dataset (Figure 3A). Pathway abundance followed a long‐tail distribution in which a small number of highly abundant pathways dominated functional profiles. Core functional analysis identified 196 pathways present in ≥ 90% of samples, collectively representing 89.6% of the total predicted pathway abundance (Figure 3B). This stands in sharp contrast to the taxonomic core, which contained no core ASVs at the ≥ 50% prevalence threshold, highlighting strong functional redundancy across highly variable microbial communities.

**FIGURE 3:**
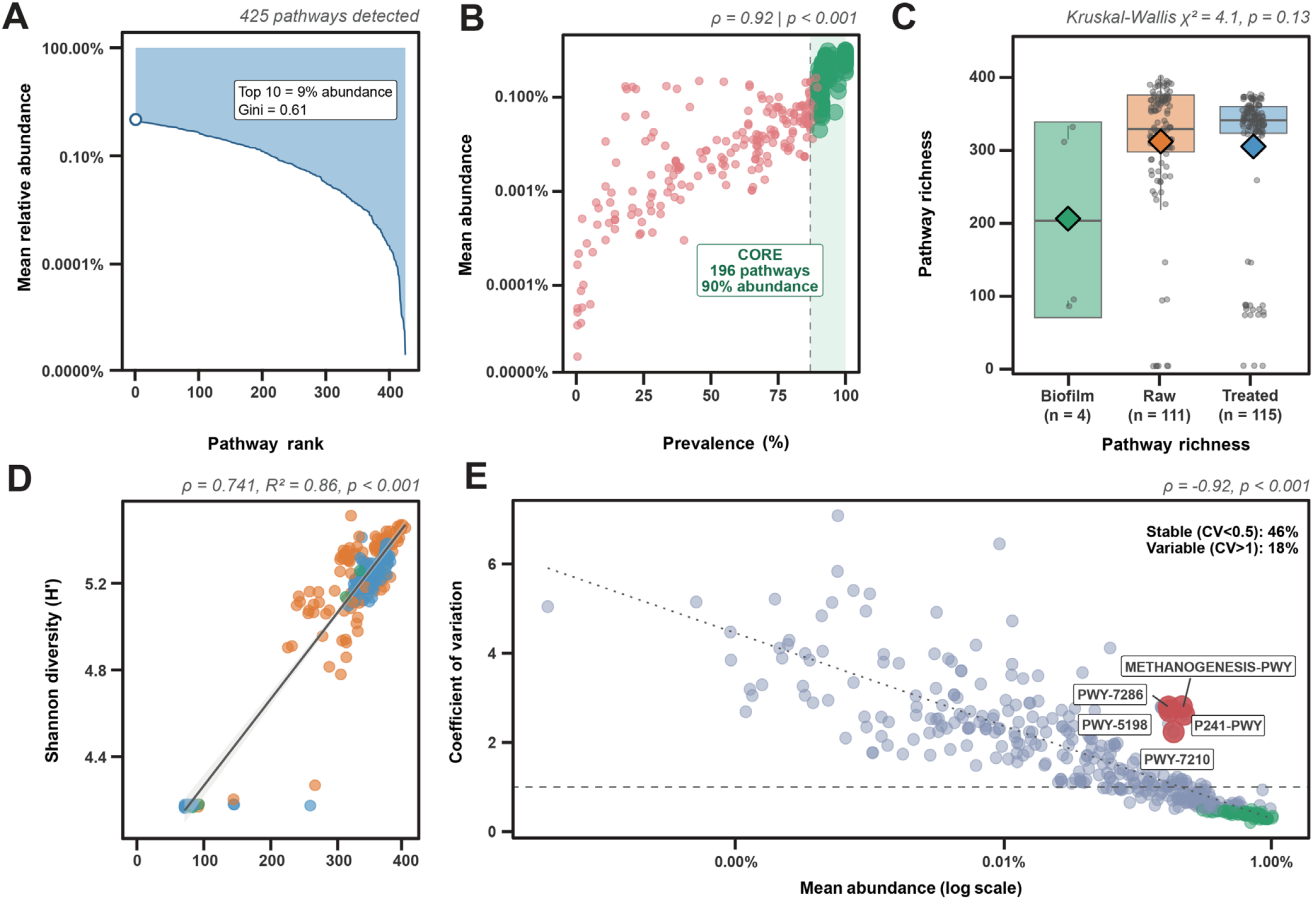
Functional pathway diversity across treatment systems. (A) Rank–abundance curve of 425 predicted MetaCyc pathways. (B) Relationship between pathway prevalence and mean relative abundance; 196 pathways present in ≥ 90% of samples comprised ~90% of total predicted functional abundance. (C) Pathway richness across sample origins (raw wastewater, treated wastewater and biofilm), with no significant differences detected (Kruskal–Wallis, *p* = 0.132). (D) Correlation between pathway richness and Shannon diversity across samples (Spearman *ρ* = 0.74), with points coloured by sample origin. (E) Coefficient of variation (CV) plotted against mean relative abundance for all pathways. Colours indicate variability classes: Stable pathways (CV < 0.5), moderately variable pathways (0.5 ≤ CV ≤ 1.0) and highly variable pathways (CV > 1.0). Approximately 46% of common pathways were classified as stable.

Functional richness and pathway abundance exhibited substantial variability across samples. The top 20 pathways ranged from approximately 0.82%–1.02% in mean relative abundance and were nearly ubiquitous, with most pathways showing 100% prevalence and low coefficients of variation (CV < 0.5), indicating high functional stability across reactors, sample types and geographic locations. Representative abundant pathways included FERMENTATION‐PWY, P125‐PWY, P42‐PWY, and PWY‐4984, all of which remained consistently enriched across samples (Table S3). Pathways associated with amino acid and carbohydrate metabolism (e.g., SER‐GLYSYN‐PWY, PWY‐5177, PWY‐5659) also showed high prevalence and low variability, further reflecting the conserved nature of core metabolic processes in wastewater treatment microbiomes.

Functional diversity patterns differed moderately across sample origins (Figure 3C). Pathway richness did not differ significantly among sample types (Kruskal–Wallis *χ* ^2^ = 4.1, df = 2, *p* = 0.132), although biofilm samples showed lower and more variable richness (median 199 pathways, IQR 88.8–312) compared with raw wastewater (median 325 pathways) and treated wastewater (median 337 pathways). Shannon diversity of pathways showed a strong positive correlation with pathway richness (Spearman *ρ* = 0.741, *p* < 0.001) (Figure 3D), indicating that samples with more pathways also tend to have more even distributions of functional potential. This strong coupling between pathway richness and evenness suggests that functional diversity closely mirrors taxonomic diversity across dairy wastewater treatment systems. Pathway variability was strongly negatively correlated with mean relative abundance (Spearman *ρ* = −0.92, *p* < 0.001; Figure 3E), indicating that more abundant predicted pathways exhibited lower variability across samples. Approximately 46% of pathways were classified as stable (CV < 0.5), whereas 18% were highly variable (CV > 1.0). These results highlight the greater stability of abundant metabolic pathways, further supporting the presence of a conserved functional repertoire across dairy wastewater microbiomes.

### 3.5 Treatment‐Specific Functional Enrichment

Kruskal‐Wallis differential abundance analysis identified 330 MetaCyc pathways that differed significantly across sample types (biofilm, raw wastewater and treated wastewater; FDR < 0.05) (Figure 4A). Raw wastewater contained the largest number of enriched pathways (166), followed by treated wastewater (62) and biofilm samples (11) (Figure 4B). These results indicate that dairy wastewater undergoes substantial functional restructuring during treatment, with distinct metabolic capabilities characterising each stage of processing.

**FIGURE 4:**
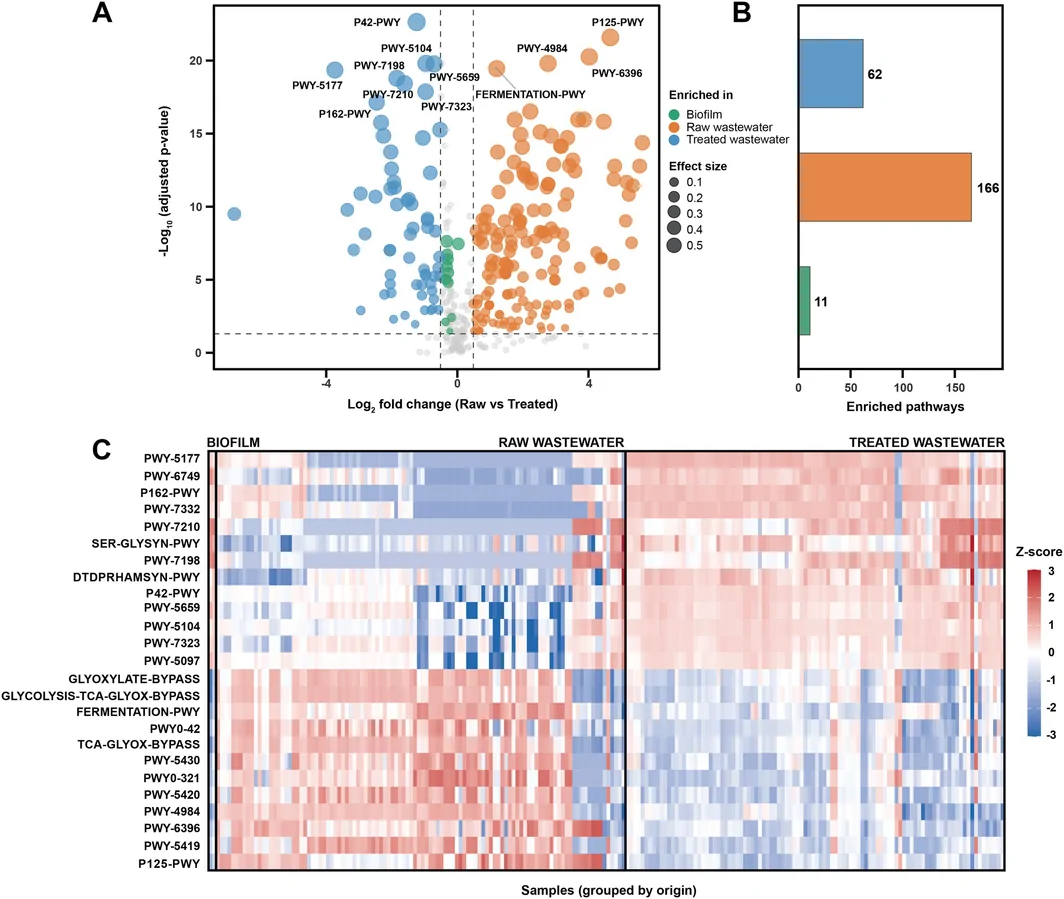
Differential pathway abundance across sample origins. (A) Volcano plot of differentially abundant pathways coloured by enrichment in treated (blue), raw (orange), or biofilm (green) samples. (B) Number of enriched pathways per origin. (C) Heatmap of top 25 differential pathways (*Z*‐score normalised).

Hierarchical clustering of differentially abundant pathways separated samples into three major functional groups corresponding to biofilm, raw wastewater and treated wastewater (Figure 4C). Pathways enriched in raw wastewater were dominated by fermentation and carbohydrate metabolism, consistent with the high organic load and diverse microbial inputs present prior to treatment. Treated wastewater exhibited enrichment in pathways associated with nitrogen metabolism, biosynthesis and detoxification, reflecting the metabolic specialisation that develops during biological treatment. Biofilm‐enriched pathways, though fewer in number, included several fermentation and amino acid metabolism processes, highlighting the distinct metabolic niches that arise within surface‐attached microbial communities.

Representative pathways illustrate these functional contrasts (Figure 5). FERMENTATION‐PWY showed elevated abundance in biofilm samples relative to raw and treated wastewater, indicating specialised fermentative activity in biofilm microenvironments. P125‐PWY (superpathway of guanosine nucleotide biosynthesis) was most abundant in raw wastewater, consistent with active microbial growth in nutrient‐rich influent. P42‐PWY (incomplete reductive TCA cycle) was enriched in biofilms, supporting anaerobic carbon fixation in oxygen‐limited layers. PWY‐4984 (urea cycle) reached highest abundance in treated wastewater, reflecting enhanced nitrogen turnover during downstream processing. Additional pathways included PWY‐5104, PWY‐5177, PWY‐5659, PWY‐6396, and SER‐GLYSYN‐PWY, each showing distinct enrichment patterns across sample origins and collectively illustrating the functional diversification that emerges across treatment stages.

**FIGURE 5:**
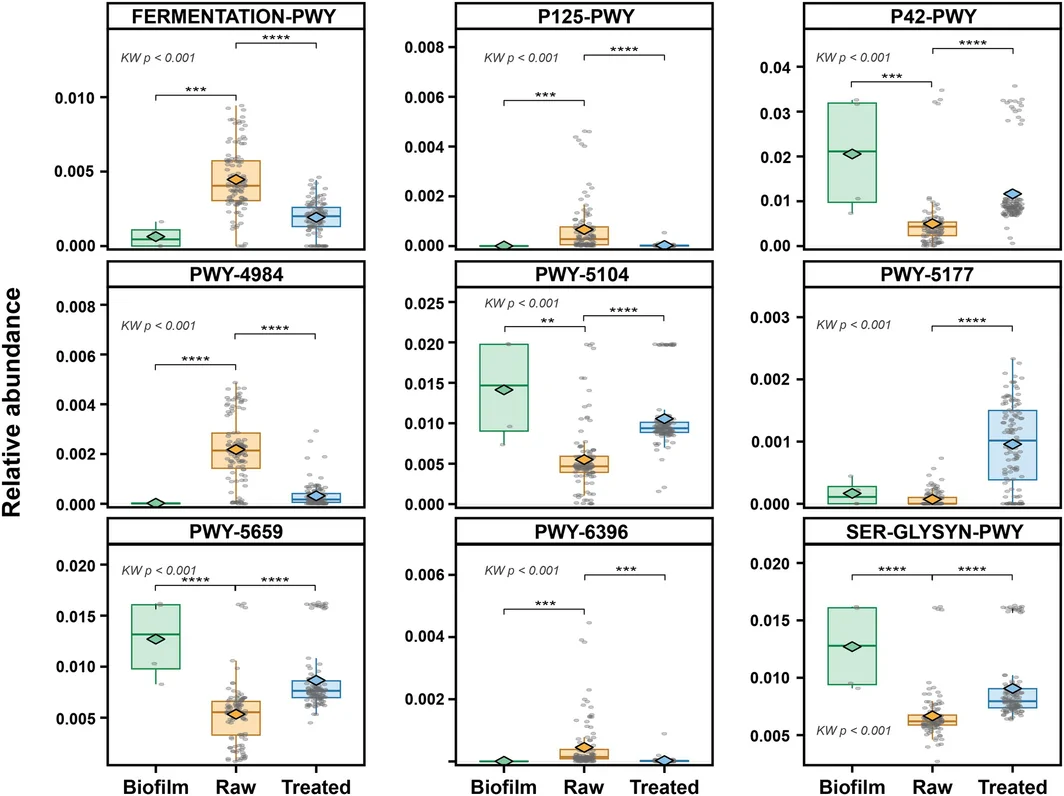
Representative differentially abundant pathways. Relative abundance of nine key pathways across sample origins. Diamonds indicate means. Kruskal‐Wallis *p* values and Dunn's post hoc comparisons shown (**p < 0.01, ***p < 0.001, ****p < 0.0001).

Together, these results demonstrate that, despite extreme taxonomic variability and minimal taxonomic core, dairy wastewater treatment systems maintain a large and conserved functional repertoire. This functional redundancy enables stable treatment performance across diverse operational configurations, environmental conditions, and geographic locations.

## 4 Discussion

### 4.1 Functional Redundancy Underlies Treatment Stability

Dairy wastewater treatment systems showed striking decoupling between taxonomic composition and functional potential. Although no ASVs were present in at least half of all 239 samples, the functional core was large and highly conserved: 196 MetaCyc pathways were detected in ≥ 90% of samples, collectively representing around 90% of all predicted functional abundance. This contrast highlights a fundamental feature of mixed microbial treatment systems, where metabolic functions remain stable even when the underlying taxa vary extensively across space, reactors and operating conditions (Louca, Polz, et al. 2018). Similar patterns have been reported in ocean (Galand et al. 2018), soil (Chen et al. 2022), and engineered microbial communities (Miki et al. 2014), where function is retained despite pronounced taxonomic turnover, but empirical demonstrations across geographically diverse wastewater treatment systems have been scarce.

The large and stable functional core indicates that essential metabolic capabilities are widely distributed across diverse taxa, supporting the ecological concept of functional redundancy. In these systems, many organisms carry genes for the same pathways, providing ‘functional insurance’ against taxonomic loss (Wang et al. 2025). The stability analysis of the most abundant pathways further supports this interpretation: nearly half of the common pathways displayed low temporal and spatial variability (CV < 0.5), despite spanning samples from psychrophilic lagoons, mesophilic reactors, and multi‐stage treatment trains across six countries. This stability reflects the centrality of certain metabolic functions, such as biosynthesis, fermentation, amino acid metabolism, and energy generation, to the operation of anaerobic and facultative treatment systems (Allison and Martiny 2008). In contrast, a subset of pathways displayed higher variability across environments, suggesting condition‐dependent metabolic responses related to nutrient availability, redox gradients, or reactor configuration.

The strong positive correlation between pathway richness and Shannon diversity (*ρ* = 0.741) suggests that samples with broader functional repertoires also tend to display more even distributions of predicted pathway abundance. This pattern is consistent with a functional architecture in which diversity extends beyond a shared core through variation in accessory functions. In microbial communities, functional richness has been linked primarily to the presence or absence of accessory genes, whereas abundance‐based variation is more strongly associated with shifts in core functions (Zhang et al. 2019). Together, these results support the interpretation that more functionally diverse communities may also occupy a broader metabolic space, consistent with the concept of greater niche breadth and metabolic flexibility (Muller 2019).

This robust functional stability helps explain why dairy wastewater treatment systems consistently achieve high performance despite fluctuations in influent composition, temperature, or microbial colonisation (Hai et al. 2014; Paulo et al. 2020). When metabolic pathways are redundantly encoded, loss or turnover of particular taxa does not eliminate critical functions, because other lineages can compensate (Barbosa Da Costa et al. 2020). This resilience mechanism contributes to the global success of biological wastewater treatment, where functional guilds, rather than specific taxa, determine system behaviour. Our findings build on this framework by showing that functional redundancy is not only present but dominant across mixed dairy wastewater systems, despite differences in geography, reactor design and operational conditions.

The taxonomic patterns observed here reinforce this interpretation. Although no ASVs were core, several higher‐level groups (3 genera, 11 families, 12 orders, 10 classes and 11 phyla) were consistently present across the dataset. Log_2_ fold enrichment analysis confirmed that these core families are differentially associated with the functional pathways corresponding to their assigned metabolic guilds (Figure S3): fermentative families such as Streptococcaceae and Carnobacteriaceae showed the strongest positive enrichment for fermentation pathways, while nitrogen‐cycling families such as Rhodocyclaceae were specifically enriched for urea cycle and nitrogen metabolism pathways. This hierarchical structure suggests that ecological niches are conserved at coarse taxonomic scales, but the organisms occupying those niches differ among studies. For example, members of the Christensenellaceae family (with different genera already described for their potential to degrade complex organic compounds (Gao et al. 2022)) were frequently detected across multiple studies, but individual ASVs were study‐specific, illustrating how functional roles can be shared by distinct local populations. Such patterns are typical of open and dynamic microbial systems, where immigration, selection, and drift continually reshape community composition without disrupting core metabolic functions (Evans et al. 2017; Justice et al. 2017).

Together, these findings show that dairy wastewater treatment is driven by a resilient and widely shared functional repertoire, even as taxonomic profiles vary substantially across reactors, countries, and environmental contexts. This functional redundancy likely underpins the stability, adaptability, and overall reliability of biological treatment technologies used worldwide.

### 4.2 Extracting Biological Signals From Meta‐Analysis Data

Meta‐analyses of microbiome datasets are inherently challenged by strong batch effects (Duvallet et al. 2017; Gibbons et al. 2018), and this pattern was evident in our analysis, where study identity explained more than half of the observed community variance (PERMANOVA *R* ^2^ = 51.2%, *p* < 0.001). This magnitude of study‐driven structure is consistent with other cross‐study microbial analyses and reflects a combination of technical and contextual factors, including DNA extraction protocols, sequencing platforms, sample preservation approaches, reactor design, and site‐specific environmental conditions (Wang and LêCao 2020). Traditional ordination methods normally cannot reliably disentangle these confounding influences from genuine ecological patterns, making it difficult to identify generalizable drivers of community structure across heterogeneous datasets (Ling et al. 2022). Variation partitioning on the 160‐sample complete‐case subset further tempered this interpretation: while marginal *R* ^2^ values attributed 51.2% of variation to study identity, only 5.28% was uniquely attributable to study‐level effects, comparable to the unique contributions of operational (5.36%) and environmental (1.75%) blocks. Most explained variance was shared across predictor blocks, indicating that study identity in meta‐analyses is not simply a technical artefact but also an aggregate proxy for the operational and environmental choices that co‐vary with who designed and ran each study. This distinction matters because it reframes batch effects as partially legitimate biological signal bundled with technical variation, rather than noise to be removed completely and not interpreted (Briscoe et al. 2022; Ma et al. 2023; Stein‐O'Brien et al. 2018).

To address this limitation, we employed generalised linear latent variable models (GLLVMs), a model‐based ordination framework for high‐dimensional data (Tang et al. 2025). Compared with constrained ordination methods such as canonical correspondence analysis or redundancy analysis, GLLVMs directly model taxon counts under a negative binomial distribution, avoiding transformation and distance computation steps that can distort sparse compositional data (Warton et al. 2012). Compared with multivariate abundance models, GLLVMs simultaneously estimate latent axes of unobserved variation within a single unified model, rather than treating ordination and hypothesis testing as separate steps (Hui et al. 2024). In contrast to non‐metric dimensionality reduction methods (NMDS, UMAP, or t‐SNE), GLLVMs produce axes with well‐defined statistical properties, including model selection criteria (AIC) for comparing competing hypotheses about which environmental drivers structure the community (Niku et al. 2019).

Critically for a multi‐study dataset, GLLVMs allow study identity to be incorporated as a fixed effect, explicitly absorbing batch structure rather than confounding it with genuine ecological gradients, while estimating the influence of environmental predictors (Korhonen et al. 2024). Model performance improved markedly when temperature and pH were included (ΔAIC = −1815 relative to the unconstrained model), and the study‐corrected model provided the best fit overall (ΔAIC = −4770). These improvements show that meaningful biological signals persist even within highly confounded multi‐study datasets, and that appropriately structured models can recover these signals despite overwhelming batch structure. Importantly, the gradients associated with temperature and pH remained significant and non‐collinear in the study‐corrected ordinations, demonstrating that they represent genuine ecological filters rather than artefacts of study‐specific conditions.

The PERMANOVA results further reinforced the contribution of biological and operational factors to community structuring. Reactor type (*R* ^2^ = 28.5%), effluent type (21.7%), temperature (17.3%), treatment configuration (17.0%), and sample origin (11.8%) each explained significant portions of variance. Although the combined explained variance exceeds 100% due to expected collinearity among operational variables, the consistent significance of these predictors across permutation tests highlights their ecological and engineering relevance. Together, these findings illustrate that it is possible to extract broadly applicable biological signals from multi‐study datasets when statistical models explicitly account for study‐level heterogeneity.

A particularly illustrative example of study‐specific structure was PRJNA826074 (Logan et al. 2022), which consistently formed a tight and isolated cluster in all community analyses. This study represents a multi‐stage treatment system with combined physical, chemical, and biological steps, but its distinguishing feature is the dominance of attached‐growth and biofilm‐associated processes. Across this dataset, community composition exhibited extremely low dispersion, and neither alpha diversity nor beta dispersion differed across sample origins (raw wastewater, treated wastewater, biofilm). More than 250 genera were unique to this single study, and no genus was shared between PRJNA826074 and at least one other study at a ≥ 50% prevalence threshold. These patterns are characteristic of attachment‐driven systems where biofilm formation on carrier materials minimises microbial washout and maximises cell retention (Abera et al. 2024; Cayetano et al. 2022). In such systems, the physical microenvironment becomes insulated from short‐term fluctuations in influent composition, leading to stable, self‐reinforcing consortia with strong internal consistency but reduced taxonomic overlap with suspended‐growth systems (Langer et al. 2014; Neeraj and Nikhil 2025).

In this context, the distinctiveness of PRJNA826074 is not an analytical outlier but an ecological consequence of adhesion‐driven microbial retention, which produces stable, mature, and highly specialised communities. Incorporating study identity into the GLLVM framework allowed this attachment‐driven structure to be accounted for rather than confounding global ecological patterns. Once this internal cohesion was modelled appropriately, temperature and pH gradients still emerged as significant ecological drivers across the broader dataset, confirming that genuine biological signal exists even when individual studies exhibit exceptionally strong system‐specific structure.

### 4.3 Environmental Drivers of Taxonomic Composition Within Functional Guilds

While functional redundancy ensures that core metabolic capabilities are preserved across treatment systems, environmental conditions determine which taxa occupy these conserved functional niches. In our dataset, temperature and pH exerted significant and independent effects on community composition, explaining 2.6% and 8.3% of genus‐level variance, respectively. Although modest compared with the influence of study identity, these gradients represent coherent ecological filters that drive predictable taxonomic shifts without disrupting the underlying functional structure of the microbial community.

Temperature acted as a strong selective force within fermentative guilds. Psychrophilic and mesophilic systems showed high presence of genera such as *Anaerostignum, Pseudoramibacter, Pseudopelobacter*, and the *Anaeromusa–Anaeroarcus* group, all of which participate in lactate and acetate turnover, butyrate formation (Andersen et al. 2015; Scarborough et al. 2018; Zhu et al. 2017), and, in some cases, medium‐chain fatty acid synthesis via reverse β‐oxidation (Liu et al. 2020; Roghair et al. 2018; Scarborough et al. 2018). These lineages declined systematically with increasing temperature, suggesting that their metabolic roles are assumed by thermotolerant functional equivalents in warmer systems. This pattern exemplifies how environmental selection reshapes the taxonomic membership of fermentative guilds while preserving their core metabolic outputs (Lawson et al. 2015; Tripathi et al. 2019), a hallmark of functional redundancy in anaerobic treatment systems.

pH imposed a second major selective gradient, structuring acidogenic versus proteolytic fermenters in opposite directions. Low‐pH environments favoured lactic acid bacteria such as *Weissella, Leuconostoc, Paucilactobacillus*, and *Bifidobacterium*, many of which enter treatment systems through dairy influents and reinforce acidic microenvironments through lactic acid production (Akpınar Kankaya and Tuncer 2022; Huang et al. 2025; Saidi et al. 2020). In contrast, alkaline conditions favoured proteolytic and amino‐acid–fermenting lineages including *Proteiniphilum, Acidaminococcus, Phascolarctobacterium, Fermentimonas* and *Treponema*. These taxa support anaerobic degradation of proteins and amino acids, producing ammonia and volatile fatty acids characteristic of alkaline fermentation. Several taxa, such as *Acidaminococcus*, displayed multidimensional responses, decreasing with temperature while increasing with pH, highlighting the complexity of ecological niches shaped by interacting physicochemical gradients (Mohidin et al. 2025).

These coordinated shifts illustrate how environmental conditions reorganise the taxonomic composition of functional guilds without altering the fundamental metabolic processes they mediate. Acidogenic and proteolytic fermenters continue to fulfil complementary roles in carbon turnover across all systems, but the specific taxa fulfilling these roles vary predictably along environmental gradients (Crognale et al. 2021; Zheng et al. 2013). Thus, process stability emerges not from taxonomic stability but from the persistence of functionally equivalent guilds capable of maintaining essential metabolic capacities across a broad range of treatment conditions.

### 4.4 Taxonomic Variability Within Functional Guilds

Although environmental gradients shape the broad structure of functional guilds, the taxonomic composition within those guilds varies widely among studies. This pattern is consistent with the absence of a universal ASV‐ or genus‐level core across the full dataset, yet cross‐study comparisons still revealed coherent ecological signals that reinforce the principle of functional redundancy. Several genera showed strong study‐specific or influent‐related patterns, indicating that local conditions determine which taxa occupy conserved metabolic niches. *Lactococcus* and *Streptococcus*, two lactic acid–producing genera commonly associated with dairy matrices (Biçer et al. 2025; Naeem et al. 2024), were among the most prevalent taxa in several studies, reaching near‐universal detection in some but remaining rare or absent in others. Their patchy distribution reflects the influence of influent composition: studies with raw dairy inputs or high lactose concentrations exhibited high prevalence of these lactic acid bacteria, whereas systems receiving synthetic or highly pretreated influent showed little to no representation. Their presence signals active fermentative turnover of simple carbohydrates and early‐stage acidification, a process that shapes downstream anaerobic degradation (Derzelle et al. 2005; Thompson and Thomas 1977). This ‘source‐derived’ signature explains why these genera appear prominently in specific datasets but are not globally conserved (Abendroth et al. 2017; Wang et al. 2016).

*Acinetobacter* displayed the most extreme variability across studies, achieving 100% prevalence in four datasets yet remaining entirely undetected in others. This genus is known for broad ecological versatility, with strains capable of thriving in nutrient‐rich wastewater, soil, sludge and aquatic environments (Qin et al. 2021). Its inconsistent distribution in our dataset aligns with its dual lifestyle: *Acinetobacter* can persist as a resilient planktonic scavenger or establish surface‐associated biofilms depending on reactor configuration, hydraulic conditions and substrate availability (Cai et al. 2021; Gangola et al. 2024). Systems with high turbulence, abundant oxygen exposure during pre‐treatment, or surfaces conducive to adhesion tended to show strong enrichment, whereas more strictly anaerobic or influent‐limited systems did not. These dynamics demonstrate how a metabolically flexible genus can function as both a transient coloniser and a persistent biofilm former depending on process design (Li et al. 2019; Sánchez‐Arroyo et al. 2024).

Members of Christensenellaceae were among the few taxa detected across a wide range of studies, showing prevalence from 21% to 100% in five of the nine datasets. Their consistent appearance in systems operating under anaerobic or microaerophilic conditions highlights their tight association with fermentative and acetogenic processes (Hankel et al. 2022; Parera Olm et al. 2025). Members of the family Christensenellaceae are strictly anaerobic, slow‐growing organisms (Ignatyeva et al. 2024) that often participate in syntrophic interactions with other fermenters and hydrogenotrophic partners (Ghouri et al. 2025). Their persistence therefore depends heavily on environmental conditions that support syntrophy, such as stable redox conditions, high organic loading and long biomass retention times (Baek et al. 2016; Gómez Camacho et al. 2019; Zhuang et al. 2015). Their occurrence across multiple studies, yet absence from others, illustrates that they represent a process‐specific rather than a universal core: their presence reflects the establishment of mature anaerobic consortia rather than a globally shared taxonomic signature.

A complementary alpha‐diversity result helps refine this interpretation. PRJNA667972, studying untreated dairy wastewater, showed the lowest median Shannon diversity in the dataset (1.41), lower than most treated‐system studies. This indicates that raw influent is not necessarily the most diverse stage of the whole treatment process. Instead, dairy wastewater may already enter treatment after strong upstream selection, potentially reflecting the composition of dairy effluents, which are typically rich in lactose, fats, proteins, and other organic compounds and may also contain detergents and sterilising agents derived from processing and cleaning operations (Joshiba et al. 2019; Singh et al. 2024). In that sense, influent diversity does not change monotonically with treatment stage. These diversity patterns were robust to normalisation choice, as rarefaction produced near‐identical study‐level and sample‐level results.

### 4.5 Treatment Stage Succession and Functional Specialisation

Treatment stage transitions were accompanied by predictable shifts in both taxonomic composition and functional potential, reflecting the sequential metabolic demands of dairy wastewater degradation. Raw wastewater samples were enriched in classical fermentative pathways, consistent with the transformation of lactose and other readily available carbohydrates into short‐chain fatty acids (Kong et al. 2008). These early‐stage communities hosted the highest relative abundance of mixed‐acid fermentation (FERMENTATION‐PWY) and nucleotide biosynthesis pathways (P125‐PWY), along with TCA‐related variants, highlighting their role in initiating the breakdown of labile substrates. This pattern reflects the dominance of fast‐growing fermentative consortia that thrive under high organic load and rapidly convert simple substrates into metabolic intermediates for downstream processes (Yin et al. 2022).

As treatment progressed and substrate availability declined, biofilm‐associated communities exhibited a clear shift toward biosynthesis, cellular maintenance and specialised fermentative functions. These communities were enriched in amino acid biosynthesis and related metabolic pathways, indicating that attached biomass supports a more metabolically diverse and resource‐efficient assemblage (Tonanzi et al. 2021). Biofilm formation likely reinforces this pattern by retaining slow‐growing organisms, stabilising syntrophic partnerships, and generating spatially structured microenvironments in which nutrient gradients and redox stratification favour metabolic specialisation (Chai et al. 2012). The enrichment of amino acid and one‐carbon metabolism pathways in biofilms further suggests an increased emphasis on recycling of cellular components and maintenance under nutrient‐limited conditions (Wei et al. 2023).

The concurrent enrichment of FERMENTATION‐PWY in biofilms, despite the dominance of fermentative metabolism in raw wastewater, reflects a difference in metabolic baseline rather than a contradiction. Raw wastewater supports fermentation as part of a broad repertoire driven by rapid turnover of labile substrates, whereas biofilms concentrate fermentative pathways within a narrower and more specialised profile shaped by syntrophic partnerships and redox stratification (Douglas 2020; Großkopf et al. 2016; Meyer et al. 2013; Yang et al. 2020). The parallel enrichment of the incomplete reductive TCA cycle (P42‐PWY) in biofilms supports this interpretation, consistent with anaerobic carbon fixation in oxygen‐limited layers coupled to fermentative carbon flow (Hessler et al. 2024; Hirose et al. 2019).

Treated wastewater contained the broadest functional repertoire, with 62 enriched pathways, representing the most metabolically differentiated stage. These communities showed consistent enrichment in the urea cycle (PWY‐4984) (Zhang et al. 2023) and in pathways linked to degradation of recalcitrant substrates, including aromatic compounds (PWY‐5104, PWY‐5177) (Alkhatib et al. 2025; Colatriano et al. 2018). This shift reflects the increasing dominance of slow‐growing, metabolically specialised guilds capable of scavenging and mineralizing complex or low‐concentration substrates that persist after primary fermentation (Fierer et al. 2007; Ma et al. 2021). The expansion of these functions aligns with the expectation that late‐stage treatment communities occupy oligotrophic niches where efficiency, substrate affinity, and metabolic complementarity drive community assembly.

These functional successions reveal that treatment robustness is maintained not by the persistence of specific taxa but by the orderly reorganisation of functional guilds across stages (Vuono et al. 2016). Despite high taxonomic turnover, the pathway‐level signatures of early fermentation, biofilm specialisation, and late‐stage substrate scavenging were remarkably consistent across studies. This demonstrates that functional transitions represent deterministic responses to substrate availability, redox structure, and biomass retention rather than idiosyncratic study effects. Consequently, deviations from expected pathway profiles, for example, reduced fermentation in raw influent or loss of specialised degradation functions in biofilms, may serve as early indicators of process imbalance (Guo and Vanrolleghem 2014; Zhou et al. 2022). A function‐centred perspective therefore provides a mechanistic foundation for interpreting treatment performance and diagnosing system disturbances.

### 4.6 Implications for Monitoring and Process Optimisation

The strong decoupling between taxonomic variability and functional stability observed in this study has important implications for monitoring and optimising dairy wastewater treatment. The persistence of a large, highly conserved functional backbone (196 pathways present in at least 90% of samples and representing almost 90% of total predicted abundance) indicates that functional redundancy underpins treatment resilience (Liu et al. 2020, 2023). This redundancy allows communities to reorganise taxonomically after environmental perturbations, such as pH shifts or substrate fluctuations, while maintaining the metabolic capacities necessary for treatment continuity.

Although shotgun metagenomics remains the most direct method for evaluating functional potential, our results demonstrate that amplicon‐based functional inference captures stable and ecologically meaningful patterns. Because many key wastewater functions are widely distributed across bacterial lineages, predicted pathway profiles generated from 16S rRNA enable the inference of metabolic potential even from compositional data (Liu et al. 2023; Matchado et al. 2024).

Such predictions offer an accessible way to infer shifts in community function without requiring high‐cost genomic sequencing, but the complementary method used to validate them needs to match the biological scale of the question. qPCR assays targeting individual functional genes (e.g., *narG, nirS, nirK*, or *ppk1*) remain useful for tracking specific enzymatic steps in nitrogen and phosphorus cycling (Hui et al. 2024; Nogales et al. 2002; Ortiz‐Severín et al. 2015; Smith et al. 2015), but aren't a strong fit for validating pathway‐level or guild‐level signals of the kind described here. Primer specificity selects for a narrow subset of sequence variants, whereas the biological signal of interest in a functional‐redundancy framework is, by definition, distributed redundantly across phylogenetically distinct taxa. cDNA‐based 16S rRNA metabarcoding could provide an appropriate complement in this context. By sequencing rRNA rather than rDNA, it profiles the putatively active fraction of the community (Blazewicz et al. 2013), preserving the taxonomic breadth of amplicon sequencing while adding an activity‐aware layer at a fraction of the cost of shotgun metatranscriptomics. Notably, 24% of the samples in the present meta‐analysis (*n* = 58) were generated from cDNA, indicating that activity‐aware amplicon sequencing is already established in the dairy wastewater literature. A direct DNA‐versus‐cDNA comparison across studies where both pools were sequenced would be a natural follow‐up, testing whether active‐community signals recapitulate the pathway‐level patterns identified here or reveal divergences invisible to DNA‐based profiling.

These findings support a shift from taxon‐centric monitoring toward frameworks grounded in functional ecology and system resilience. Managing wastewater systems as adaptive microbial ecosystems requires prioritising the environmental and operational conditions that sustain functional guilds rather than attempting to maintain specific taxa. Integrating 16S‐based functional prediction with quantitative gene assays and operational metadata would enable the development of predictive diagnostics that identify system deterioration earlier and more reliably than taxonomic indicators. Such a function‐centred paradigm aligns with broader principles of ecological engineering, emphasising process stability through the maintenance of functional capacity rather than compositional uniformity (Allison and Martiny 2008; Chen et al. 2024; Juan‐García et al. 2017).

### 4.7 Limitations and Future Directions

Several limitations must be considered when interpreting the present findings. First, amplicon‐based functional inference estimates potential rather than expressed activity. PICRUSt2 predicts the gene content of the community from 16S rRNA sequences and reference genomes; it does not measure whether those genes are transcribed or whether the encoded enzymes are active under reactor conditions (Douglas et al. 2020; Toole et al. 2021). Because uncultured organisms were detected in 8 of the 9 studies at 100% prevalence, substantial metabolic novelty likely exists that is not represented in reference databases, leading to potential underestimation or misrepresentation of pathway content (Agrawal et al. 2019). Complementary approaches, including cDNA‐based 16S metabarcoding (activity‐aware), shotgun metatranscriptomics (transcriptome‐scale activity), and metagenomics (pangenome‐resolved potential) (Rosconi et al. 2022), would be important to confirm that the conserved pathway backbone identified here corresponds to a conserved functional output rather than a conserved repertoire of gene presence (Bharti and Grimm 2021; Shaffer et al. 2022).

Second, the dataset is taxonomically and operationally uneven, which constrains the scope of the conclusions. Biofilm samples (*n* = 4) represent less than 2% of the whole dataset, so pathway enrichment specific to biofilms should be interpreted as preliminary, and replication in dedicated biofilm‐focused studies is required. Geographic coverage is skewed, with 48.3% of samples originating from Ireland; patterns that appear as global in this study partly reflect Irish operational practices. Two studies used synthetic wastewater rather than industrial dairy effluent, thereby simplifying the substrate composition relative to real‐world influents (Bharti and Grimm 2021). Third, the meta‐analysis lacks paired treatment‐performance metadata for most samples. Effluent quality metrics such as COD removal efficiency, total nitrogen removal, phosphorus removal, and methane yield were either unreported or reported in non‐comparable formats across the original studies. As a result, the conserved pathway backbone identified here could be correlated with process outcomes only indirectly, through treatment‐stage labels (raw, treated, biofilm).

Direct linkage between functional potential and measurable treatment performance remains one of the most substantial open questions and the single most important target for future cross‐study standardisation efforts. Similarly, operational drivers known to affect microbial composition, such as hydraulic retention time, organic loading rate, reactor hydrodynamics, and the presence of chemical inhibitors, were unevenly reported and largely confounded with study identity, limiting their separability in the GLLVM and PERMANOVA frameworks (Gibbons et al. 2018; Wang and LêCao 2020).

Finally, our analysis focused on bacterial communities, while other microbial domains, including methanogenic archaea, fungi, protozoa, and viruses, are integral to treatment processes (Shaffer et al. 2022). Multi‐domain analyses incorporating these groups would yield a more complete picture of functional redundancy and microbial assembly mechanisms in dairy wastewater systems. Future work should prioritise validating predicted functions through metagenomics and metatranscriptomics, experimentally manipulating functional guilds to test causal relationships, linking pathway profiles to treatment performance metrics such as COD removal or methane production, and developing function‐based diagnostics for operational monitoring (Liu et al. 2022; Płaza et al. 2021; Vítězová et al. 2020). These efforts will deepen our understanding of how functional redundancy and environmental filtering jointly shape microbial ecosystems in engineered environments and will facilitate the design of more resilient and predictable biological treatment processes.

## 5 Conclusion

This study demonstrates that dairy wastewater treatment systems exhibit extensive taxonomic variability but maintain a highly conserved functional core. Although no ASVs and few genera were shared across studies, nearly 200 MetaCyc pathways were consistently present and accounted for most predicted metabolic activity. Temperature and pH influenced community composition, but their effects were relatively small compared with differences among studies and reactor designs. Despite this heterogeneity, functional profiles reorganised in predictable ways across treatment stages, reflecting stable metabolic roles associated with fermentation, biosynthesis and substrate degradation. These findings show that functional redundancy, rather than taxonomic stability, supports the resilience and performance of dairy wastewater treatment. Approaches that track functional indicators and key metabolic genes may therefore provide more reliable diagnostics of system stability and operational health than taxonomic composition alone.

## Author Contributions

**Alex Batista Trentin:** methodology, investigation, software, formal analysis, visualization, writing – original draft, writing – review and editing. **Sara Letícia Stolfo dos Santos:** conceptualization, data curation, writing – original draft. **André Felipe Bendix:** writing – review and editing, writing – original draft. **Laila Cristina Lopes:** writing – original draft, writing – review and editing. **Thaís Fernandes Mendonça Mota:** writing – review and editing. **Samara Silva de Souza:** writing – review and editing. **Nédia de Castilhos Ghisi:** writing – review and editing. **Deborah Catharine De Assis Leite:** writing – review and editing, writing – original draft, conceptualization, methodology, data curation, supervision, investigation, validation, visualization, funding acquisition, project administration, resources.

## Funding

This work was supported by Coordenação de Aperfeiçoamento de Pessoal de Nível Superior and Universidade Tecnológica Federal do Paraná.

## Conflicts of Interest

The authors declare no conflicts of interest.

## Supporting information


**Figure S1:** PRISMA‐style study selection. Flow diagram of the literature search and screening process.

**Figure S2:** Analytical workflow. Four‐phase pipeline applied to all retained studies. Phase 1: database search and study selection. Phase 2: study‐specific DADA2 denoising naïve Bayes taxonomy against SILVA 138.2, and SEPP placement into backbone to enable cross‐region comparison (V1–V2, V3–V4, V4–V5). Phase 3: filtering in R v4.3/phyloseq v1.44. Phase 4: parallel taxonomic analyses (alpha diversity, core microbiome, GLLVM, PERMANOVA) and functional analyses (PICRUSt2 pathway prediction, core functional repertoire, Kruskal–Wallis differential abundance with *η* ^2^ effect sizes and BH‐FDR correction).

**Figure S3:** Core taxonomic groups and their associated functional pathways. (A) Prevalence of 13 core taxa detected in ≥ 50% of samples, annotated by primary functional guild (literature‐based). Families are shown in the upper panel, genera in the lower panel. (B) Log_2_ fold enrichment of MetaCyc pathway abundance in samples where each core family is detected versus absent. Pathways are grouped by functional category. Red indicates enrichment, blue indicates depletion.

**Table S1:** Complete metadata for all samples included in the analysis. Full standardised metadata for the 239 samples used in this study, including study identifier, sample origin, effluent type, reactor type, treatment category, temperature regime, pH, sequencing type (DNA or cDNA), and all other operational and contextual variables extracted from publications and BioSample records.
**Table S2:** Core microbiome summary across taxonomic ranks. Number and proportion of taxa meeting the core threshold (≥ 50% prevalence across samples) at each taxonomic rank. No ASVs were shared across ≥ 50% of samples, while core representation increased at higher taxonomic levels.
**Table S3:** PERMANOVA results for environmental and operational predictors of community structure. Results of permutational multivariate analysis of variance (PERMANOVA) evaluating the contribution of Study, Reactor type, Effluent type, Temperature regime, Treatment type, and Sample origin to variation in genus‐level community composition (*n* = 239). Study identity explained the largest proportion of variance (*R* ^2^ = 51.2%, *p* = 0.001), followed by reactor configuration and effluent type. Full statistics include degrees of freedom, sum of squares, *R* ^2^, *F*‐statistic and *p* values.
**Table S4:** Differential abundance of MetaCyc pathways across sample origins. Results of Kruskal–Wallis tests evaluating differences in predicted pathway abundance among raw wastewater, treated wastewater and biofilm samples. Shown are *χ* ^2^ statistics, degrees of freedom, *p* values, FDR‐adjusted *p* values and effect sizes (*η* ^2^).

## Data Availability

The data that support the findings of this study are available on request from the corresponding author. The data are not publicly available due to privacy or ethical restrictions.
