# Supplementary material for: Functional Redundancy Enables Stable Treatment Performance Across Taxonomically Diverse Dairy Wastewater Microbiomes
Source: Environ Microbiol Rep. 2026 Oct 20;18(5):e70448. doi: 10.1111/1758-2229.70448 (PMC13682855; doi:10.1111/1758-2229.70448)
Supplement: Supplementary file 1 [file EMI4-18-e70448-s001.pdf]

Figure S3. Core taxonomic groups and their associated functional pathways

(A) Prevalence of 13 core taxa detected in ≥50% of samples, annotated by primary functional guild (literature-based). Families are shown in the upper panel, genera in the lower panel. (B) Log_2 fold enrichment of MetaCyc pathway abundance in samples where each core family is detected versus absent. Pathways are grouped by functional category. Red indicates enrichment, blue indicates depletion.

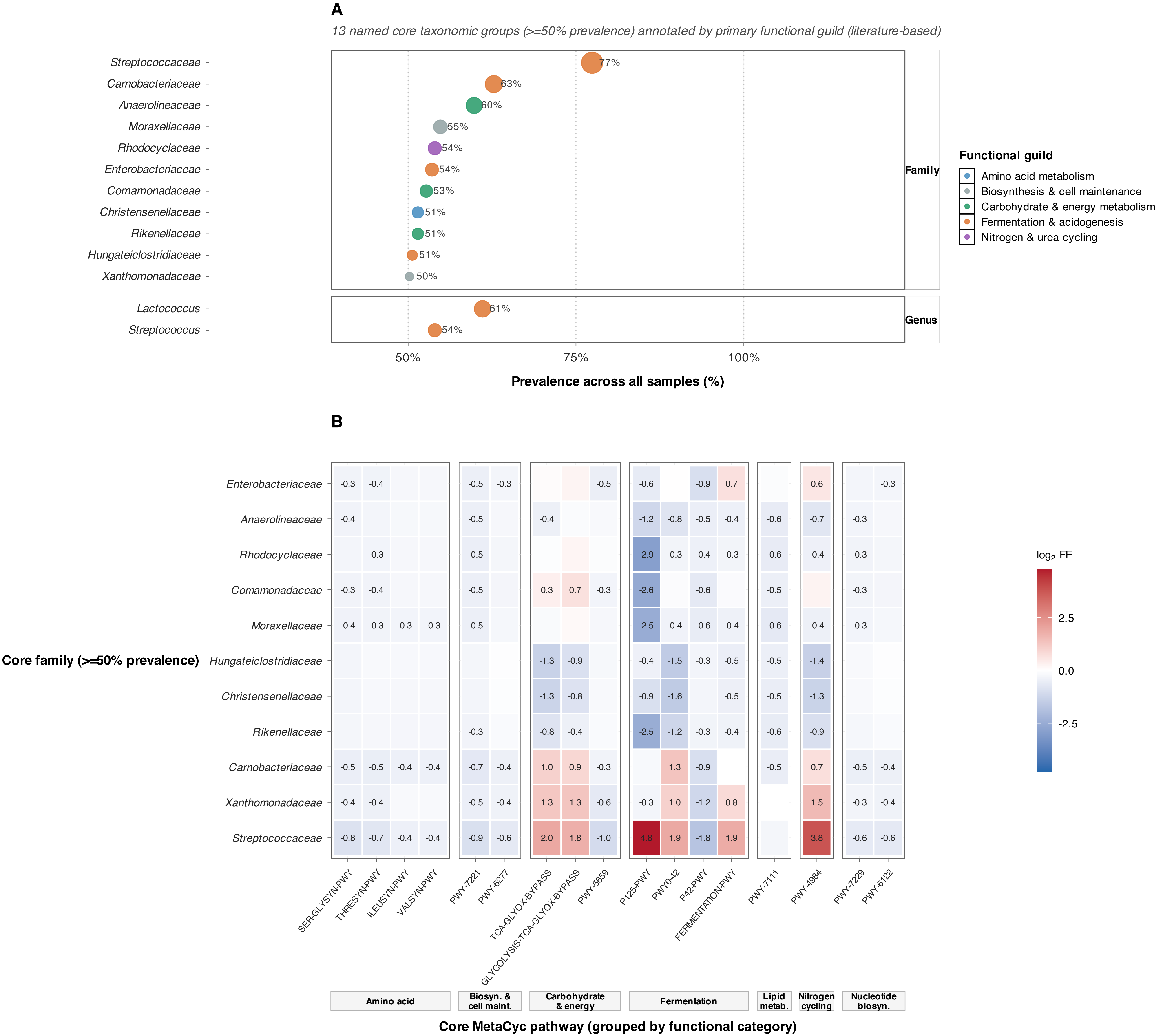

	SER-GLYSYN-PWY	THRESYN-PWY	ILEUSYN-PWY	VALSYN-PWY	PWY-7221	PWY-6277	TCA-GLYOX-BYPASS	GLYCOLYSIS-TCA-GLYOX-BYPASS	PWY-5659	P125-PWY	PWY0-42	P42-PWY	FERMENTATION-PWY	PWY-7111	PWY-4984	PWY-7229	PWY-6122
Enterobacteriaceae	-0.3	-0.4			-0.5	-0.3			-0.5	-0.6		-0.9	0.7		0.6		-0.3
Anaerolineaceae	-0.4				-0.5		-0.4			-1.2	-0.8	-0.5	-0.4	-0.6	-0.7	-0.3	
Rhodocyclaceae		-0.3			-0.5					-2.9	-0.3	-0.4	-0.3	-0.6	-0.4	-0.3	
Comamonadaceae	-0.3	-0.4			-0.5		0.3	0.7	-0.3	-2.6		-0.6		-0.5		-0.3	
Moraxellaceae	-0.4	-0.3	-0.3	-0.3	-0.5					-2.5	-0.4	-0.6	-0.4	-0.6	-0.4	-0.3	
Hungateiclostridiaceae							-1.3	-0.9		-0.4	-1.5	-0.3	-0.5	-0.5	-1.4		
Christensenellaceae							-1.3	-0.8		-0.9	-1.6		-0.5	-0.5	-1.3		
Rikenellaceae					-0.3		-0.8	-0.4		-2.5	-1.2	-0.3	-0.4	-0.6	-0.9		
Carnobacteriaceae	-0.5	-0.5	-0.4	-0.4	-0.7	-0.4	1.0	0.9	-0.3		1.3	-0.9		-0.5	0.7	-0.5	-0.4
Xanthomonadaceae	-0.4	-0.4			-0.5	-0.4	1.3	1.3	-0.6	-0.3	1.0	-1.2	0.8		1.5	-0.3	-0.4
Streptococcaceae	-0.8	-0.7	-0.4	-0.4	-0.9	-0.6	2.0	1.8	-1.0	4.8	1.9	-1.8	1.9		3.8	-0.6	-0.6
