# Supplementary material for: Functional Redundancy Enables Stable Treatment Performance Across Taxonomically Diverse Dairy Wastewater Microbiomes
Source: Environ Microbiol Rep. 2026 Oct 20;18(5):e70448. doi: 10.1111/1758-2229.70448 (PMC13682855; doi:10.1111/1758-2229.70448)
Supplement: Supplementary file 2 [file EMI4-18-e70448-s003.pdf]

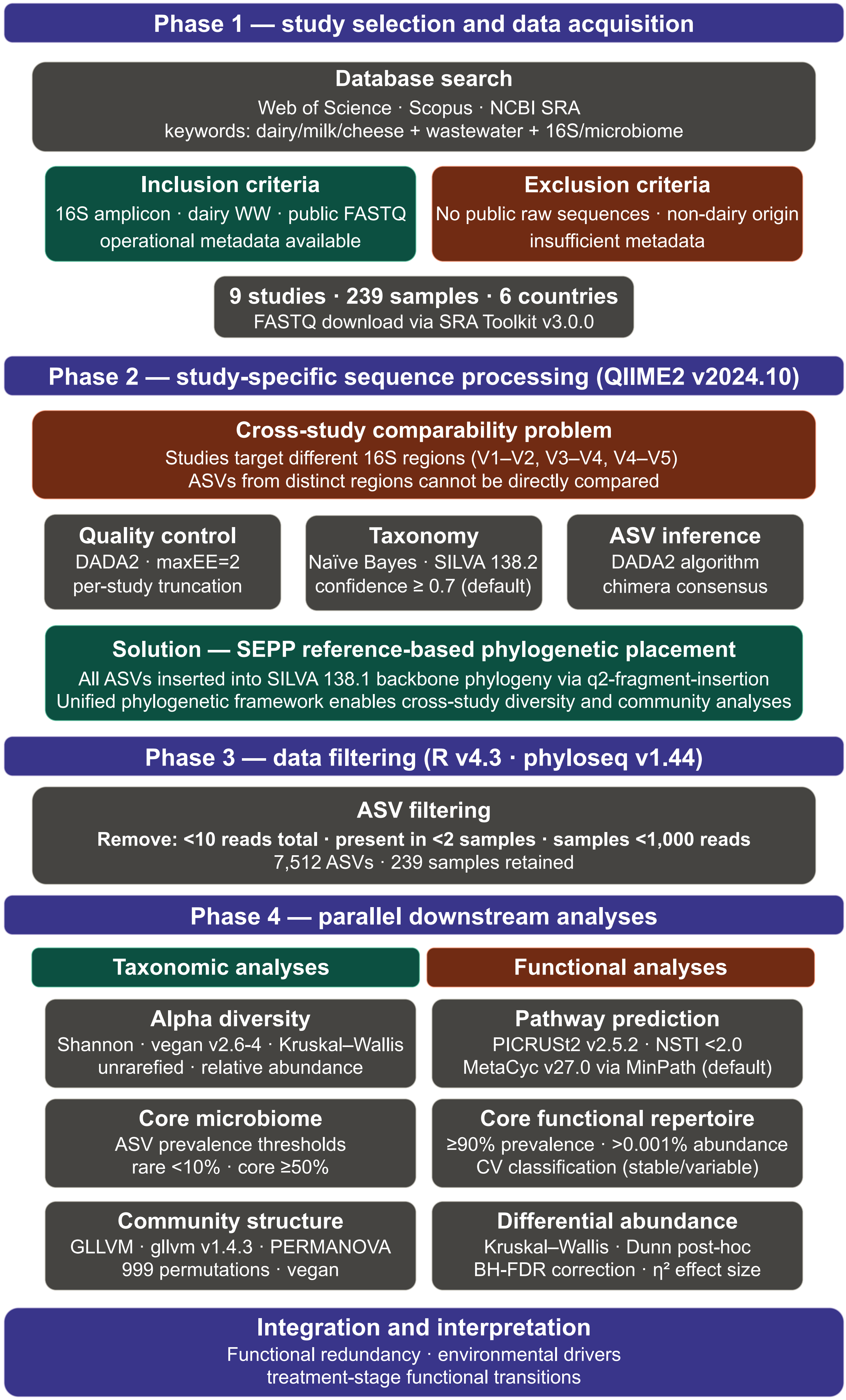
Phase 1 — study selection and data acquisition
Database search
Web of Science · Scopus · NCBI SRA
keywords: dairy/milk/cheese + wastewater + 16S/microbiome
Inclusion criteria
16S amplicon · dairy WW · public FASTQ
operational metadata available
Exclusion criteria
No public raw sequences · non-dairy origin
insufficient metadata
9 studies · 239 samples · 6 countries
FASTQ download via SRA Toolkit v3.0.0
Phase 2 — study-specific sequence processing (QIIME2 v2024.10)
Cross-study comparability problem
Studies target different 16S regions (V1–V2, V3–V4, V4–V5)
ASVs from distinct regions cannot be directly compared
Quality control
DADA2 · maxEE=2
per-study truncation
Taxonomy
Naïve Bayes · SILVA 138.2
confidence ≥ 0.7 (default)
ASV inference
DADA2 algorithm
chimera consensus
Solution — SEPP reference-based phylogenetic placement
All ASVs inserted into SILVA 138.1 backbone phylogeny via q2-fragment-insertion
Unified phylogenetic framework enables cross-study diversity and community analyses
Phase 3 — data filtering (R v4.3 · phyloseq v1.44)
ASV filtering
Remove: <10 reads total · present in <2 samples · samples <1,000 reads
7,512 ASVs · 239 samples retained
Phase 4 — parallel downstream analyses
Taxonomic analyses
Functional analyses
Alpha diversity
Shannon · vegan v2.6-4 · Kruskal–Wallis
unrarefied · relative abundance
Pathway prediction
PICRUSt2 v2.5.2 · NSTI <2.0
MetaCyc v27.0 via MinPath (default)
Core microbiome
ASV prevalence thresholds
rare <10% · core ≥50%
Core functional repertoire
≥90% prevalence · >0.001% abundance
CV classification (stable/variable)
Community structure
GLLVM · gllvm v1.4.3 · PERMANOVA
999 permutations · vegan
Differential abundance
Kruskal–Wallis · Dunn post-hoc
BH-FDR correction · η^2 effect size
Integration and interpretation
Functional redundancy · environmental drivers
treatment-stage functional transitions
