# Supplementary material for: Functional Redundancy Enables Stable Treatment Performance Across Taxonomically Diverse Dairy Wastewater Microbiomes
Source: Environ Microbiol Rep. 2026 Oct 20;18(5):e70448. doi: 10.1111/1758-2229.70448 (PMC13682855; doi:10.1111/1758-2229.70448)
Supplement: Supplementary file 3 [file EMI4-18-e70448-s004.pdf]

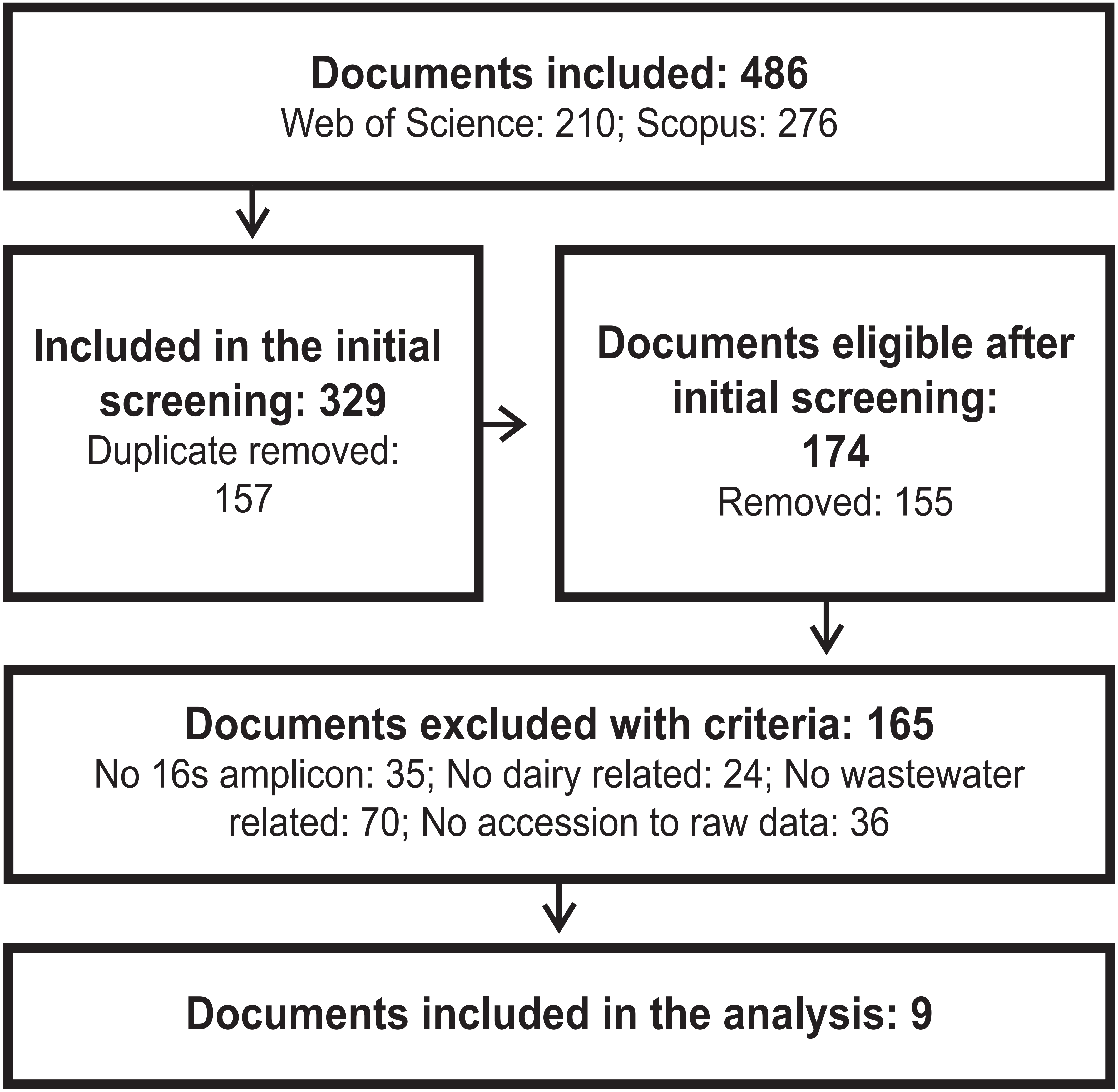
Documents included: 486
Web of Science: 210; Scopus: 276
Included in the initial screening: 329
Duplicate removed: 157
Documents eligible after initial screening: 174
Removed: 155
Documents excluded with criteria: 165
No 16s amplicon: 35; No dairy related: 24; No wastewater related: 70; No accession to raw data: 36
Documents included in the analysis: 9
